# Modes of transmission and attack rates of group A Streptococcal infection: a protocol for a systematic review and meta-analysis

**DOI:** 10.1186/s13643-021-01641-5

**Published:** 2021-03-31

**Authors:** Dylan D. Barth, Jessica Daw, Ruomei Xu, Stephanie Enkel, Janessa Pickering, Tracy McRae, Mark E. Engel, Jonathan Carapetis, Rosemary Wyber, Asha C. Bowen

**Affiliations:** 1 Wesfarmers Centre for Vaccines and Infectious Diseases, Telethon Kids Institute, Perth, Western Australia Australia; 2 The University of Western Australia, Perth, Western Australia Australia; 3 AFROStrep Registry, Department of Medicine, The University of Cape Town, Cape Town, South Africa; 4 Department of Infectious Diseases, Perth Children’s Hospital, Perth, Western Australia; 5 The George Institute for Global Health, Sydney, New South Wales Australia

**Keywords:** Group A *Streptococcus*, Transmission, Systematic review, Environmental health, Public health

## Abstract

**Background:**

Group A *Streptococcus* (Strep A) is an important cause of mortality and morbidity globally. This bacterium is responsible for a range of different infections and post-infectious sequelae. Summarising the current knowledge of Strep A transmission to humans will address gaps in the evidence and inform prevention and control strategies. The objective of this study is to evaluate the modes of transmission and attack rates of group A streptococcal infection in human populations.

**Methods:**

This systematic review protocol was prepared according to the Preferred Reporting Items for Systematic reviews and Meta-analysis Protocols (PRISMA-P) 2015 Statement. Using a comprehensive search strategy to identify any transmission studies that have been published in English since 1980, full-text articles will be identified and considered for inclusion against predefined criteria. We will include all studies reporting on Strep A transmission, who have identified a mode of transmission, and who reported attack rates. Risk of bias will be appraised using an appropriate tool. Our results will be described narratively and where feasible and appropriate, a meta-analysis utilizing the random-effects model will be used to aggregate the incidence proportions (attack rates) for each mode of transmission. In addition, we will also evaluate the *emm* genotype variants of the M protein causing Strep A infection and the association with transmission routes and attack rates, if any, by setting, socioeconomic background and geographical regions.

**Discussion:**

We anticipate that this review will contribute to elucidating Strep A modes of transmission which in turn, will serve to inform evidence-based strategies including environmental health activities to reduce the transmission of Strep A in populations at risk of severe disease.

**Trial registration:**

Systematic review registration: PROSPERO (CRD42019138472).

**Supplementary Information:**

The online version contains supplementary material available at 10.1186/s13643-021-01641-5.

## Background

*Streptococcus pyogenes*, also known as Group A *Streptococcus* (Strep A), is responsible for a wide range of diseases and is in the top 10 pathogens globally causing morbidity and mortality, particularly in disadvantaged settings around the world [1]. Strep A causes superficial infections (pharyngitis, impetigo, scarlet fever), invasive infections (cellulitis, skeletal infections, sepsis, necrotising fasciitis, and toxic shock syndrome), and post-infectious immune-mediated sequelae (acute rheumatic fever/rheumatic heart disease, post-streptococcal glomerulonephritis). The prevalence of rheumatic heart disease is estimated to be 33 million (95% confidence interval (CI), 29.7 million to 43.1 million) cases globally and the cause of 319,000 (95% CI, 297,000 to 337,300) deaths each year [2]. Given the unavailability of a vaccine, treatment options for Strep A infections rely on antibiotics, predominantly penicillin [3–5]. There are > 220 different Strep A *emm* types classified according to the amino acid sequence of M proteins found on the bacterial cell wall [6]. Epidemiological studies have reported an association of *emm* type with different disease manifestations, populations and climate [1, 7–11].

Modes of transmission for pathogens are classified as contact (both direct and indirect), droplet or airborne. Vehicle or biological vectors may also play a role in disease transmission [12]. Traditionally, transmission of Strep A has been primarily attributed to large respiratory droplets [13–18] based on studies that used one of two methods: (a) evaluating the saliva of patients who had sore throat and scarlet fever, and (b) environmental epidemiology which involved measuring the quantity of Strep A discharged into the air in a controlled room through coughing, sneezing and talking. More recently, a combination of methodological approaches including culture from biological swabs, environmental swabs, and environmental settle plates have elucidated additional modes of transmission. These include nasal secretions, sputum or spit (droplet) [19, 20], small airborne particles, e.g. dust (airborne) [20–24], skin-to-skin contact (direct contact) [19, 25–27], surfaces (indirect contact) [28–30], bedding and fabrics (indirect contact) [31–33], food (vehicle) [34, 35], and insects (biological vectors) [36–38]. Both the infectious and carrier state may lead to transmission, dominated by the infectious state [39, 40]. A few studies have reported Strep A transmission from carriers to uninfected individuals who have subsequently become symptomatic [41–44].

In addition to the pathogen characteristics and mode of transmission, some settings may influence the risk of Strep A infection. The household setting, which provides an extended duration of exposure to individuals with Strep A, is associated with an increased risk of transmission and subsequent infection [44–46]. Close household contacts of patients with invasive Strep A infection have approximately 2000 times the risk of also developing invasive Strep A infection, most noticeable in mother-neonate pairs and cohabiting partners > 74 years old [47]. Infection with Strep A in households is associated with an increased risk of post-streptococcal sequelae, e.g. acute rheumatic fever (ARF) and rheumatic heart disease (RHD) [48]. In a study conducted in Australia, around 9% of newly diagnosed children with ARF have a sibling with RHD [49]. Similarly, in New Zealand [50] and Uganda [51], siblings of children living with RHD were 2 and 4.5 times more likely to have RHD detected by echocardiography, respectively. Overcrowded households and limited household hygiene facilities are likely to compound these risk factors [52]. Institutional settings, including hospitals and aged care facilities, also present the risk of nosocomial spread of Strep A infections. This can be increased by lapses in infection control measures, posing a significant risk to both healthcare workers and patients [53–56].

Transmission-based precautions are a crucial component of infection control [57]. Understanding the modes of transmission of Strep A infection will allow strategies to focus on interrupting these modes of exposure and reduce the risk of disease. To date, no evidence-based synthesis has been undertaken to understand the various modes of transmission of Strep A. The objectives of this review are to (1) synthesize the evidence of modes of transmission for Strep A, (2) calculate and compare attack rates against mode of transmission, and (3) correlate *emm* types of Strep A isolated from clinical swabs and mode of transmission, where possible.

## Methods

This protocol has been written in accordance with the Preferred Reporting Items for Systematic Reviews and Meta-Analyses Protocol (PRISMA-P) guidelines (Additional file 1) [58]. The protocol is registered with PROSPERO (CRD42019138472).

### Eligibility criteria

Studies will be selected according to the following criteria: participants, study designs, condition/exposure(s), and outcome(s) of interest.

#### Inclusion criteria

1. We will include studies involving children, adolescents, and adults regardless of age, gender, health conditions, or other sociodemographic characteristics.
2. All study designs (including but not limited to case series, outbreak investigations, cross-sectional, cohort and case-control studies) investigating modes of transmission of Strep A to humans resulting in human Strep A infection (both symptomatic disease with clinical relevance to Strep A infection and asymptomatic carriage) will be included. Where available, posters and abstracts will be included separately under ‘partially published research’ to avoid publication bias and skewing of cohort results due to poor ratings in study quality assessment from the limited availability of full-text publications.
3. Studies that proposed a mode of transmission will be included, regardless of use of molecular typing to confirm the mode. Transmission modes that will be reviewed: include airborne, droplet, contact (direct, indirect), vehicle, and biological vectors.
4. Household (or familial) and healthcare worker acquisition will be included even if there is no designated mode of transmission implicated in the study.
5. Studies published in English between January 1980 and December 2019.

#### Exclusion criteria

1. Where duplicate publications are identified, the most recent version will be included.
2. Narrative reviews, opinion pieces, letters, and articles lacking primary attack rate data.
3. Laboratory based research, including that which only analysed *emm* types associated with outbreaks.
4. Articles addressing symptoms, diagnostic method, or treatment choice in outbreaks.
5. Outbreak summaries with no mention of transmission modes.
6. Articles which summarise an infection trend rather than outbreaks.

#### Condition being studied

All Strep A infections and sequelae will be assessed, including asymptomatic carriage. There will be no restrictions on the participants’ age, gender, health conditions, and demographics for inclusion.

#### Types of outcome measures

There will be two outcome measures: firstly, documented evidence of Strep A transmission by different modes; and secondly, the attack rate of people with Strep A infection (symptomatic) and detection (asymptomatic carriage). Attack rates will be calculated as the number of people who had Strep A isolated divided by the number of people at risk of becoming ill from Strep A. Attack rates between each of the mode of transmission will be compared.

#### Definitions

Modes of transmission can be both direct and indirect [12], and include:

- Airborne, e.g. infectious agents carried by fine expectorate sputum of dust suspended in air,
- Droplet, e.g. produced by sneezing, coughing, and talking.
- Contact, e.g. skin-to-skin or contact with a contaminated environment)
- Vehicles, e.g. inanimate objects that can indirectly transmit an infectious agent include food, water, bodily fluids, and fomites and
- Vectors (e.g. flies, mosquitoes, fleas which can carry an infectious agent through mechanical or biological means

### Search strategy and information sources

The primary source of literature will be a structured search strategy for PubMed (Additional file 2) and will be adapted for use in other databases including Scopus, EMBASE, Web of Science, and CINAHL. The secondary source of potentially relevant material will be a search of grey literature which includes WHO IRIS library database, Trove, Research Data Australia, the Grey Literature Report, and Australian Infection Prevention and Control. For clinical trial registries, the Cochrane Central Register of Clinical Trials, WHO International Clinical Trials Registry Platform, Australian New Zealand Clinical Trial Registry, and ClinicalTrial.gov. We will also search reference lists of included studies for any potentially relevant articles. The search will be restricted to studies published in English between January 1980 and December 2019. The literature searches will be designed and conducted by the review team with experience in developing search terms for a comprehensive search strategy.

### Screening and selection procedure

Titles and abstracts identified by the literature search will be screened for possible inclusion by two reviewers (RX/DB/JP) based on the eligibility criteria described above. Secondly, full-text articles will be evaluated for inclusion (JD/SE) with independent data extraction completed by two reviewers (JD/SE/TM) using a standardised data collection form in Microsoft Excel, with discrepancies adjudicated by a third author (AB). Thirdly, references of all considered articles will be hand-searched to identify any relevant reports missed by the search strategy.

### Data extraction

A summary of the data to be collected is shown in Table 1. We will assess the molecular techniques (e.g. typing and pulse field gel electrophoresis (PFGE)) used to confirm the mode of Strep A transmission. Data collection will be recorded in a Microsoft Excel spreadsheet and will be tested by the team before data collection will commence. Discrepancies will be resolved by discussion and consensus involving a third author. Partially published studies will have the data extraction limited to location, setting, attack rate, and proposed mode of transmission. Where data are missing or incomplete, primary authors will be contacted to seek additional data.

**Table 1:** Data to be collected in the data extraction of included studies

Information on studies	Information on participants	Information on infections
• Study author • Study year • Study design • Study setting (domestic, nosocomial or public) • Geographical region	• Age group/range • Gender • Income category • Ethnicity • Health conditions which might increase the risk of Strep A infection or carriage, e.g. immunodeficiency states (immunocompetent or immunocompromised) or underlying diseases	• Modes of Strep A transmission • Types of Strep A infections • Symptoms • Strep A *emm* strains • Time period in which transmission occurred • Season of participation • Number of people exposed (denominator) • Numbers of people with Strep A infected/detected (only primary transmission) (numerator) • Number of deaths • Asymptomatic carriage rates • Sampling method used to collect samples • Laboratory methods used to analyse samples

### Assessment of risk of bias

Risk of bias will be assessed independently by two reviewers using the Critical Appraisal tool from the Joanna Briggs Institute [59]. Each domain will be assessed as ‘yes’ or ‘no’ with a tally of the number of ‘yes’ results reflected in the total score. In addition, for the question which addresses the appropriateness of statistical analysis used in each study, we will assess whether the numerator and denominator were reported to calculate the attack rate.

### Data synthesis

Data will be reported according to modes of transmission. Where household and healthcare worker transmission studies specify only a designated method of acquisition rather than a mode of transmission, or where different strains have been identified between environmental and clinical Strep A isolates, these studies will be included for qualitative assessment and the synthesis of data will be reported according to the synthesis without meta-analysis (SWiM) guidelines [60].

Studies will be included in a meta-analysis if molecular techniques have been applied to identify common Strep A strains obtained from clinical and environmental swabs. Data will be analysed using STATA version 16 (StataCorp, College Station, TX, USA). Our analysis will calculate attack rates of Strep A infection in the respective studies, i.e. number of people infected divided by number of people exposed, which will thereafter, be subjected to meta-analysis (random-effects model, due to the expected variability across the studies) to derive summary attack rate estimates (overall pooled estimate with the pooled 95% CI). In addition, we will derive standard errors (SE) where studies have provided the numerator and denominators to calculate the attack rates for Strep A. Attack rates of the different studies will be analysed, and the pooled estimates will be conducted using the Metaprop package. The pooled rates will be estimated using the Freeman-Tukey double arcsine transformation method to stabilise the variance of attack rates within each study [61].

Heterogeneity between included studies will be assessed using the *I*^2^ heterogeneity statistic, reported as a percentage to determine the extent of variation between the studies [62]. Deeks [62] defines categories of heterogeneity with a value ≤ 25% as low, 26–50% moderate, 51–75% substantial, and 76–100% as considerable heterogeneity. Where heterogeneity is statistically significant, a sensitivity analysis will be conducted to explore potential sources of heterogeneity and to establish if the meta-analysis results are influenced by the effect of the quality of the studies (risk of bias) or sample size. Where available, a subgroup analysis will be conducted for the attack rates of Strep A in patients from different settings (nosocomial, domestic and public settings) and for different geographical regions (Northern or Southern hemisphere). There is, however, the potential that there may be significant diversity in the included studies in terms of setting, population, disease, and attack rates, so as to not render the data amenable to pooling. Should heterogeneity exist, or if attack rates are not reported or cannot be calculated, results will be discussed in a narrative review.

By using appropriate strategies, we will minimize the potential biases in the review process. Unpublished papers, grey literature, and preprints will be included in the review. Potential publication biases will be explored with graphic assessment using Funnel plots. Funnel plots are recommended when there are at least 10 studies included for meta-analysis.

## Discussion

This systematic review seeks to provide robust contemporary evidence for the modes involved in Strep A transmission. The rigorous methods to be used in this systematic review will ensure robust synthesis of available data to provide empirical evidence necessary for researchers, policy-makers, public health, and environmental health stakeholders, thus reducing the burden of all Strep A causing invasive, non-invasive, and immune-mediated diseases. Given that the burden of severe Strep A disease is concentrated in developing countries and among Indigenous populations living in developed countries, this review can help establish recommendations to reduce the disproportionate impact on marginalised populations globally. A limitation of the review is the inclusion of non-randomised and observational studies that consequently will result in a high variability across included studies.

We do not expect any protocol amendments arising. However, should any amendments be deemed necessary, it will be reported in the published review.

The results of this review will be disseminated in the form of a peer-reviewed journal article and findings will also be presented to relevant health authorities and medical services working in remote regions.

## Supplementary Information

**Additional file 1:.** (Additional file 1.pdf) contains Preferred Reporting Items for Systematic Reviews and Meta-Analyses Protocol (PRISMA-P) checklist from which this protocol was written [58].

**Additional file 2:.** (Additional file 2.pdf) contains the search strategy for use in the PubMed database.

## Data Availability

Not applicable.

## Abbreviations

Strep A: Group A *Streptococcus* (or *Streptococcus pyogenes*)
ARF: Acute rheumatic fever
RHD: Rheumatic heart disease
CI: Confidence intervals
PRISMA-P: Preferred Items for Systematic reviews and Meta-analysis Protocols
SE: Standard errors
CRE: Centre for research excellence
PFGE: Pulse field gel electrophoresis
SWiM: Synthesis without meta-analysis

## References

[1] Carapetis JR, Steer AC, Mulholland EK, Weber M. The global burden of group A streptococcal diseases. Lancet Infect Dis. 2005;5(11):685–94. 10.1016/S1473-3099(05)70267-X. 10.1016/S1473-3099(05)70267-X 16253886

[2] Watkins DA, Johnson CO, Colquhoun SM, Karthikeyan G, Beaton A, Bukhman G, et al. Global, Regional, and National Burden of Rheumatic Heart Disease, 1990-2015. N Engl J Med. 2017;377(8):713–22. 10.1056/NEJMoa1603693. 10.1056/NEJMoa1603693 28834488

[3] Bowen AC, Tong SYC, Andrews RM, O’Meara IM, McDonald MI, Chatfield MD (2014). Short-course oral co-trimoxazole versus intramuscular benzathine benzylpenicillin for impetigo in a highly endemic region: an open-label, randomised, controlled, non-inferiority trial. Lancet.

[4] Cole C, Gazewood J (2007). Diagnosis and treatment of impetigo. Am Fam Physician.

[5] Gerber MA, Baltimore RS, Eaton CB, Gewitz M, Rowley AH, Shulman ST, Taubert KA (2009). Prevention of rheumatic fever and diagnosis and treatment of acute streptococcal pharyngitis: a scientific statement from the American Heart Association Rheumatic Fever, Endocarditis, and Kawasaki Disease Committee of the Council on Cardiovascular Disease. Circulation.

[6] McMillan DJ, Dreze PA, Vu T, Bessen DE, Guglielmini J, Steer AC (2013). Updated model of group A *Streptococcus* M proteins based on a comprehensive worldwide study. Clin Microbiol Infect.

[7] May PJ, Bowen AC, Carapetis JR (2016). The inequitable burden of group A streptococcal diseases in Indigenous Australians. Med J Aust.

[8] Bowen AC, Mahé A, Hay RJ, Andrews RM, Steer AC, Tong SYC, Carapetis JR (2015). The global epidemiology of impetigo: a systematic review of the population prevalence of impetigo and pyoderma. PLoS One.

[9] Wong SS, Yuen K (2012). *Streptococcus pyogenes* and re-emergence of scarlet fever as a public health problem. Emerg Microbes Infect.

[10] Steer AC, Law I, Matatolu L, Beall BW, Carapetis JR (2009). Global emm type distribution of group A streptococci: systematic review and implications for vaccine development. Lancet Infect Dis.

[11] Towers RJ, Carapetis JR, Currie BJ, Davies MR, Walker MJ, Dougan G (2013). Extensive diversity of *Streptococcus pyogenes* in a remote human population reflects global-scale transmission rather than localised diversification. PLoS One.

[12] Bonita R, Beaglehole R, Kjellstrom T. Basic epidemiology. 2nd ed. Organisation WH, editor. 2006.

[13] Hamburger M, Robertson OH (1948). Expulsion of group A hemolytic streptococci in droplets and droplet nuclei by sneezing, coughing and talking. Am J Med.

[14] Hamilton A (1905). Dissemination of streptococci through invisible sputum: In relation to scarlet fever and sepsis. JAMA.

[15] Hamburger M (1944). Studies on the transmission of hemolytic streptoccus infections: II. Beta hemolytic streptococci in the saliva of person with positive throat cultures. J fof Infect Dis.

[16] Hamburger M (1944). Studies on the transmission of hemolytic streptococcus infections. I: Cross infections in army hospital wards. J Infect Dis.

[17] Ross PW (1971). Beta-haemolytic streptococci in saliva. J Hyg (Lond).

[18] Morawska L (2006). Droplet fate in indoor environments, or can we prevent the spread of infection?. Indoor Air.

[19] Hamburger M (1947). Transfer of beta hemolytic streptococci by shaking hands. Am J Med.

[20] Mj H, Green MJ, Hamburger VG (1945). The problem of the “dangerous carrier” of hemolytic streptococci 1: number of hemolytic streptococci expelled by carriers with positive and negative nose cultures. J Infect Dis.

[21] Brown WA, Allison VD (1937). Infection of the air of scarlet-fever wards with *Streptococcus pyogenes*. J Hyg.

[22] Lemon HM, Loosli CG, Hamburger M (1948). Transmission and control of respiratory diseases in army barracks: II. The spread of haemolytic streptococcal infections among enlisted personnel. J Fof Infect Dis.

[23] Perry WD, Siegel AC, Rammelkamp CH (1957). Transmission of group A streptococci. II. The role of contaminated dust. Am J Hyg.

[24] White E (1936). On the possible transmission of haemolytic streptococci by dust. Lancet.

[25] Ferrieri P, Dajani AS, Wannamaker LW, Chapman SS (1972). Natural history of impetigo. I. Site sequence of acquisition and familial patterns of spread of cutaneous streptococci. J Clin Invest.

[26] Hare R (1941). Haemolytic streptococci in normal people and carriers. Lancet.

[27] Colebrook L, Maxted WR, Morris JA (1935). The presence of haemolytic and other streptococci on human skin. J Pathol Bacteriol.

[28] Cruickshank JG, Lightfoot NF, Sugars KH, Colman G, Simmons MD, Tolliday J, Oakley EHN (1982). A large outbreak of streptococcal pyoderma in a military training establishment. J Hyg.

[29] Sarangi J, Rowsell R (1995). A nursing home outbreak of group A streptococcal infection: case control study of environmental contamination. J Hosp Infect.

[30] Loosli CG, Lemon HM, Wise H, Robertson OH (1948). Studies on the transmission and control of respiratory disease within Army Barracks: I. Hamolytic streptococcal contamination of the environment. J Infect Dis.

[31] Lemon HM (1997). The nasal carrier of beta-hemolytic streptococci. N Engl J Med.

[32] Perry WD, Siegel AC, Rammelkamp CH, Wannamaker LW, Marple EC (1957). Transmission of group A streptococci. I. The role of contaminated bedding. Am J Hyg.

[33] Mackintosh CA, Hoffman PN (1984). An extended model for transfer of micro-organisms via the hands: differences between organisms and the effect of alcohol disinfection. J Hyg.

[34] Greig JD, Todd ECD, Bartleson CA, Michaels BS (2007). Outbreaks where food workers have been implicated in the spread of foodborne disease. Part 1. Description of the problem, methods, and agents involved. J Food Prot.

[35] Wilson LG (1986). The historical riddle of milk-borne scarlet fever. Bull Hist Med.

[36] Bassett DC (1970). Hippelates flies and streptococcal skin infection in Trinidad. Trans R Soc Trop Med Hyg.

[37] Chifanzwa R. House Fly (Musca Domestica L.) Temporal and Spatial Immune Response to *Streptococcus Pyogenes* and *Salmonella Typhimurium*: role of pathogen density in bacterial fate, persistence and transmission. Vol. Master of, Department of Biology. Georgia Southern University; 2011.

[38] Cogen AL, Nizet V, Gallo RL (2008). Skin microbiota: a source of disease or defence?. Br J Dermatol.

[39] Yokchoo N, Patanarapeelert N, Patanarapeelert K (2019). The effect of group A streptococcal carrier on the epidemic model of acute rheumatic fever. Theor Biol Med Model.

[40] DeMuri GP, Wald ER (2014). The group A streptococcal carrier state reviewed: Still an enigma. J Pediatric Infect Dis Soc.

[41] Martin JM, Group A (2004). Streptococci Among School-Aged Children: Clinical Characteristics and the Carrier State. Pediatrics.

[42] Kaplan EL, Gastanaduy AS, Huwe BB (1981). The role of the carrier in treatment failures after antibiotic for group A streptococci in the upper respiratory tract. J Lab Clin Med.

[43] Cockerill FR, MacDonald KL, Thompson RL, Roberson F, Kohner PC, Besser-Wiek J (1997). An outbreak of invasive group A streptococcal disease associated with high carriage rates of the invasive clone among school-aged children. JAMA.

[44] Mazón A, Gil-Setas A, de la Gándara LJ S, Vindel A, Sáez-Nieto JA (2003). Transmission of *Streptococcus pyogenes* causing successive infections in a family. Clin Microbiol Infect.

[45] James WE, Badger GF, Dingle JH (1960). A study of illness in a group of Cleveland families. XIX. The epidemiology of the acquisition of group A streptococci and of associated illnesses. N Engl J Med.

[46] Musher DM (2003). How contagious are common respiratory tract infections?. N Engl J Med.

[47] Mearkle R, Saavedra-Campos M, Lamagni T, Usdin M, Coelho J, Chalker V, et al. Household transmission of invasive group A Streptococcus infections in England: A population-based study, 2009, 2011 to 2013. Eurosurveillance. 2017;22(19). 10.2807/1560-7917.ES.2017.22.19.30532 PMC5476984 28537550

[48] Karthikeyan G, Guilherme L. Acute rheumatic fever. Lancet. 2018;392(10142):161–74. 10.1016/S0140-6736(18)30999-1. 10.1016/S0140-6736(18)30999-1 30025809

[49] Noonan S, Zurynski YA, Currie BJ, McDonald M, Wheaton G, Nissen M, Curtis N, Isaacs D, Richmond P, Ramsay JM, Elliott EJ, Carapetis JR (2013). A national prospective surveillance study of acute rheumatic fever in Australian children. Pediatr Infect Dis J.

[50] Culliford-Semmens N, Tilton E, Wilson N, Stirling J, Dougherty R, Gentles T (2018). The New Zealand familial echo study reveals high prevalence of rheumatic heart disease amongst parents and siblings of children with rheumatic fever: echocardiography should be offered to first degree relatives. J Paediatr Child Health.

[51] Aliku T, Sable C, Scheel A, Tompsett A, Lwabi P, Okello E, McCarter R, Summar M, Beaton A (2016). Targeted echocardiographic screening for latent rheumatic heart disease in Northern Uganda: Evaluating Familial Risk Following Identification of an Index Case. PLoS Negl Trop Dis.

[52] Coffey PM, Ralph AP, Krause VL (2018). The role of social determinants of health in the risk and prevention of group A streptococcal infection, acute rheumatic fever and rheumatic heart disease: a systematic review. PLoS Negl Trop Dis.

[53] Daneman N, McGeer A, Low DE, Tyrrell G, Simor AE, McArthur M, Schwartz B, Jessamine P, Croxford R, Green KA, Ontario Group A Streptococcal Study Group (2005). Hospital-Acquired Invasive Group A Streptococcal Infections in Ontario, Canada, 1992-2000. Clin Infect Dis.

[54] Deutscher M, Schillie S, Gould C, Baumbach J, Mueller M, Avery C, van Beneden CA (2011). Investigation of a group A streptococcal outbreak among residents of a long-term acute care hospital. Clin Infect Dis.

[55] Thigpen MC, Richards CL, Lynfield R, Barrett NL, Harrison LH, Arnold KE (2007). Invasive group A streptococcal infection in older adults in long-term care facilities and the community, United States, 1998-2003. Emerg Infect Dis.

[56] Trell K, Jörgensen J, Rasmussen M, Senneby E (2020). Management of an outbreak of postpartum *Streptococcus pyogenes emm*75 infections. J Hosp Infect.

[57] Siegel JD, Long S, Pickering LK, Prober CG (2012). Pediatric Infection Prevention and Control. Principles and Practice of Pediatric Infectious Diseases: Part 1 Communicable diseases in children.

[58] Moher D, Shamseer L, Clarke M, Ghersi D, Liberati A, Petticrew M (2015). Preferred reporting items for systematic review and meta-analysis protocols ( PRISMA-P ) 2015 statement. Syst Rev.

[59] Moola S, Munn Z, Tufanaru C, Aromataris E, Sears K, Sfetcu R, Currie M, Qureshi R, Mattis P, Lisy K, Mu P-F. Chapter 7: Systematic reviews of etiology and risk. In: Aromataris E, Munn Z, editors. Joanna Briggs Institute Reviewer's Manual. The Joanna Briggs Institute; 2017. Available from https://reviewersmanual.joannabriggs.org/.

[60] Campbell M, McKenzie JE, Sowden A, Katikireddi SV, Brennan SE, Ellis S, et al. Synthesis without meta-analysis (SWiM) in systematic reviews: Reporting guideline. BMJ. 2020. 10.1136/bmj.l6890. 10.1136/bmj.l6890 PMC7190266 31948937

[61] Nyaga VN, Arbyn M, Aerts M (2014). Metaprop: A Stata command to perform meta-analysis of binomial data. Arch Public Heal.

[62] Deeks JJ, Higgins JP, Altman DG. Analysing data and undertaking meta‐analyses. Analysing data and undertaking meta‐analyses. In: Higgins JP, Thomas J, Chandler J, Cumpston M, Li T, Page MJ, Welch VA, editors. Cochrane Handbook for Systematic Reviews of Interventions. 2019. 10.1002/9781119536604.ch10.

