# Supplementary material for: Modes of transmission and attack rates of group A Streptococcal infection: a protocol for a systematic review and meta-analysis
Source: Syst Rev. 2021 Mar 31;10:90. doi: 10.1186/s13643-021-01641-5 (PMC8011413; doi:10.1186/s13643-021-01641-5)
Supplement: Supplementary file 2 — Additional file 2:. (Additional file 2.pdf) contains the search strategy for use in the PubMed database. [file 13643_2021_1641_MOESM2_ESM.pdf]

Additional file 2. Search strategy for PubMed database.

Search	Query
#1	("streptococcus pyogenes"[MeSH Terms] OR "streptococcus pyogenes"[Title/Abstract]) OR “S.pyogenes”[Title/Abstract] OR “Strep A”[Title/Abstract] OR ("Group A"[Title/Abstract] AND ("streptococcus" [Title/Abstract] OR "streptococci" [Title/Abstract] OR "streptococcal"[Title/Abstract])) OR (("beta-haemolytic"[Title/Abstract] OR "beta-hemolytic"[Title/Abstract]) AND ("streptococcus"[Title/Abstract] OR “streptococci”[Title/Abstract] OR “streptococcal”[Title/Abstract]))
#2	(("Disease transmission, Infectious"[MeSH Terms] OR "transmission"[Subheading] OR "transmission"[Title/Abstract] OR "transmit"[Title/Abstract] OR “transmitted”[Title/Abstract]) OR ("communicable diseases"[MeSH Terms] OR "communicable disease*"[Title/Abstract] OR ("communicable"[Title/Abstract] AND "disease*"[Title/Abstract]) OR "communicability"[Title/Abstract]) OR ("disease outbreaks"[MeSH Terms] OR "outbreak*"[Title/Abstract]) OR ("epidemics"[MeSH Terms] OR "epidemic*"[Title/Abstract]) OR ("cross infection"[MeSH Terms] OR "cross infection*"[Title/Abstract]) OR (("nosocomial"[Title/Abstract] AND "infection*"[Title/Abstract]) OR "nosocomial infection*"[Title/Abstract])) AND (("contaminate*"[All Fields] OR “contaminant*”[All Fields] OR “contamination”[All Fields]) OR ("contagion*"[All Fields] OR “contagious”[All Fields]) OR ("infection"[MeSH Terms] OR “infection*”[All Fields] OR "infect"[All Fields] OR “infected”[All Fields] OR “infectious”[All Fields]) OR ("inoculate*"[All Fields] OR “inoculation”[All Fields]) OR "contact*"[All

	Fields] OR "acquisition"[All Fields] OR "spread*"[All Fields] OR "household*"[All Fields] OR ("faecal-oral"[All Fields] OR "fecal-oral"[All Fields]) OR ("fece*"[All Fields] OR "faece*"[All Fields]) OR "stool*"[All Fields] OR "perianal"[All Fields] OR "air-borne"[All Fields] OR "water-borne"[All Fields] OR "food-borne"[All Fields] OR "vertical"[All Fields] OR "horizontal"[All Fields] OR ("cough"[All Fields] OR “coughing”[All Fields]) OR ("sneeze"[All Fields] OR “sneezing”[All Fields]) OR "nose blowing"[All Fields] OR "wound*"[All Fields] OR "trauma*"[All Fields] OR "damage*"[All Fields] OR "pus"[All Fields] OR "crust*"[All Fields] OR "ulcer*"[All Fields] OR "lesion*"[All Fields] OR "blister*"[All Fields] OR "rash*"[All Fields] OR "droplet*"[All Fields] OR "aerosol*"[All Fields] OR "oral secretion*"[All Fields] OR "nasal secretion*"[All Fields] OR "saliva"[All Fields] OR "dust"[All Fields] OR "skin"[All Fields] OR "fomites"[All Fields] OR "bedding"[All Fields] OR "clothing"[All Fields] OR "surface*"[All Fields] OR "fabric*"[All Fields])
#3	("streptococcal infections"[MeSH Terms] OR "streptococcal infection*"[Title/Abstract]) OR "pharyngitis"[All Fields] OR "sore throat"[All Fields] OR "quinsy"[All Fields] OR "tonsillitis"[All Fields] OR "throat infection*"[All Fields] OR "peritonsillar abscess"[All Fields] OR "pharyngotonsillitis"[All Fields] OR "pneumonia"[All Fields] OR ("acute lower respiratory infection*"[All Fields] OR “ALRI*”[All Fields]) OR ("lower respiratory tract infection*"[All Fields] OR “LRTI*”[All Fields]) OR "scarlet fever"[All Fields] OR "sepsis"[All Fields] OR "septic"[All Fields] OR ("bacteraemia"[All Fields] OR "bacteremia"[All Fields]) OR "toxic shock syndrome"[All Fields] OR ("necrotizing fasciitis"[All Fields] OR "necrotising

	fasciitis"[All Fields]) OR "impetigo"[All Fields] OR "skin sore*"[All Fields] OR "pyoderma"[All Fields] OR "skin infection*"[All Fields] OR "dermatitis"[All Fields] OR "cellulitis"[All Fields] OR "erysipelas"[All Fields] OR "skeletal infection*"[All Fields] OR "osteomyelitis"[All Fields] OR "septic arthritis"[All Fields] OR "bone infection*"[All Fields] OR "joint infection*"[All Fields] OR "rheumatic fever"[All Fields] OR (“rheumatic heart disease*”[All Fields] OR “RHD*”[All Fields]) OR "acute post streptococcal glomerulonephritis"[All Fields] OR "meningitis"[All Fields] OR "puerperal sepsis"[All Fields] OR "puerperal fever"[All Fields] OR "pyogenic infection*"[All Fields]
#4	#1 AND #2 AND #3 (+ filters activated: publish year: 1980-2019, English, human)
#5 (for exclusion criteria)	“antioxidant*”[Title] OR “biofilm*”[Title] OR “bactericidal”[Title] OR “exotoxin*”[Title] OR “streptolysin*”[Title] OR “zoonotic”[Title] OR “adhesion”[Title] OR “coloni*ation”[Title] OR “probiotic”[Title] OR (“mouse”[Title] OR “model*”[Title]) OR “bacteriophage*”[Title] OR “polymerase*”[Title] OR “transcriptome*”[Title] OR “neutrophil*”[Title] OR “mass spectrometry”[Title] OR “zooepidemicus”[Title] OR “pyrogenic”[Title] OR “adhesion*”[Title] OR “collagen*”[Title] OR “phage*”[Title] OR “polymorphism”[Title] OR “platelet*”[Title] OR “epitope*”[Title] OR “prophage*”[Title] OR “epithelial cell*”[Title] OR “rapid diagnostic testing”[Title] OR “aptamer”[Title] OR “point-of-care test*”[Title] OR “dose-response model*”[Title] OR “genomics”[Title] OR “fluorescent”[Title] OR “ERIC-PCR”[Title] OR “synthetase*”[Title] OR “restriction enzyme*”[Title] OR “agglutination testing”[Title] OR “esterase*”[Title] OR “vitro”[Title] OR

	“host cell receptor*”[Title] OR “amino acid*”[Title] OR (“IgA”[Title] OR “IgG”[Title] OR “IgM”[Title]) OR “hybridi*ation”[Title] OR “host-pathogen interaction*”[Title] OR “internali*ation”[Title] OR “membrane*”[Title] OR “fimbriae”[Title] OR “electrophoresis”[Title] OR “polymorphic”[Title] OR “fibroblast*”[Title] OR “toxin*”[Title] OR “superantigen*”[Title] OR “bioluminescence”[Title] OR “lipoglycopeptide*”[Title] OR “luminex”[Title] OR “intrahost”[Title] OR “peptide*”[Title] OR “pharmacokinetic*”[Title] OR “nanoparticle*”[Title] OR “lactoferrin*”[Title] OR “siderophore*”[Title] OR “methyltransferase*”[Title] OR “fluoroquinolone”[Title] OR “epithelium”[Title] OR “pentagloblin*”[Title] OR “prophage*”[Title] OR “capsular”[Title]
#6	#4 NOT #5
